# 27 years of experience with the Fontan procedure: characteristics and clinical outcomes of children in a tertiary referral hospital

**DOI:** 10.1186/s13019-023-02148-x

**Published:** 2023-01-18

**Authors:** Ahmet Bülent Polat, Murat Ertürk, Ozan Uzunhan, Nur Karademir, Kazım Öztarhan

**Affiliations:** 1 Department of Cardiovascular Surgery, Florence Nightingale Hospital, T.C. Demiroglu Bilim University, İzzetpaşa Mah, Abide-I Hürriyet Cad, No:166, Sisli, 34394 Istanbul, Turkey; 2 Department of Newborn, Florence Nightingale Hospital, T.C. Demiroglu Bilim University, İzzetpaşa Mah, Abide-I Hürriyet Cad, No:166, Sisli, 34394 Istanbul, Turkey; 3 Florence Nightingale Hospital, T.C. Demiroglu Bilim University, İzzetpaşa Mah, Abide-I Hürriyet Cad, No:166, Sisli, 34394 Istanbul, Turkey; 4 Department of Pediatric Cardiology, Florence Nightingale Hospital, T.C. Demiroglu Bilim University, İzzetpaşa Mah, Abide-I Hürriyet Cad, No:166, Sisli, 34394 Istanbul, Turkey

**Keywords:** Fontan operation, Lateral tunnel, Extracardiac condiut, Glenn shunt, BT shunt

## Abstract

**Background:**

The Fontan operation has improved the survival of children born with congenital heart disease with single ventricle physiology. The most widely adopted variations of the Fontan procedure are the extracardiac conduit, the lateral tunnel ve the intra/extracardiac conduit with fenestration. Despite advances in the treatment and prevention of early and late complications that may develop after Fontan surgery, morbidity still remains an important problem.

**Methods:**

304 patients who underwent Fontan surgery in our center between 1995 and 2022 were included in our study. The complications that developed in patients who underwent primary Fontan or lateral tunnel surgery and extracardiac conduit Fontan application were compared.

**Results:**

Classic Fontan surgery and lateral tunnel surgery were performed in 26 of the patients, and extracardiac Fontan surgery was performed in 278 patients. 218 of 304 cases were patients with single ventricular pathology. 86 cases were patients with two ventricular morphologies but complex cardiac pathology. Fenestration was performed in only 6 patients, other patients did not require fenestration. The mean follow-up period of our patients was 12 years (3 months–27 years). When the complications between Fontan procedures were compared in our study, it was found that the length of hospital stay and mortality were statistically significantly reduced in patients who underwent extracardiac Fontan surgery. There was no significant difference in terms of complications that can be seen after Fontan surgery and the length of stay in the intensive care unit.

**Conclusion:**

Fontan complex is a palliative surgery for children with complex heart disease. Palliative surgical operations aimed at the preparation of the Fontan circulation lead to the preparation of the pulmonary vascular bed and the preservation of ventricular function. The techniques applied in Fontan surgery affect the early and long-term complications and the survival of the patients. In our study, when we examined the patients who extracardiac conduit Fontan procedure for the non-cardiac route, we found that mortality and morbidity were minimal.

## Introductıon

The beginning of Fontan surgery in 1968 marked a turning point in the treatment and prognosis of the most complex congenital heart diseases [1]. The Fontan procedure provides a palliative treatment option for pediatric patients with functionally single ventricle congenital heart disease, with an estimated incidence of 0.08 to 0.4 per 1000 live births [2].

Today, it is a preferred palliative surgery method in congenital heart diseases with single ventricular anatomy and physiology and in other complex groups that do not allow biventricular repair (unbalanced ventricular, atrioventricular septal defect (AVSD), double outlet right ventricle, whole correction). possibilities, complex transposition of the great arteries, etc.). This surgical technique distinguishes between pulmonary and systemic venous return [3].

The original approach was a Glenn shunt, which describes patients with tricuspid atresia and was first applied by Fontan et al. [1] in 1968, where the superior vena cava is connected to the right pulmonary artery. Few changes have been made since then. Shortly after Fontan, Kreutzer et al. [4] described modified atriopulmonary connection techniques. The right atrial-right ventricular connection was reported by Björk et al. [5] in 1979. The total cavopulmonary connection (TCPC) or lateral tunnel, which is still widely practiced, has started to be used by Laval et al. [6] in 1988.

Fontan operation is performed not only in cardiac malformations with single ventricular morphology, but also in almost all cardiac malformations that are not suitable to biventricular repair. Early and late death rates are constantly decreasing,owing to advances in surgical technique and perioperative care, the development of preoperative selection criteria, and innovations in medical and interventional treatment strategies [7–10].

There was a clear decrease in early death rates after the fontan procedure.The early death rate, which was 20.1% in the first studies, decreased to 0.5% in the latest publications [11–15]. Decrease in systemic ventricular systolic and diastolic function, increase in pulmonary vascular resistance, development of systemic venous thrombus, protein-losing enteropathy, lymphatic dysfunction, development of arrhythmia, liver dysfunction are important in the long-term performance of the Fontan circulation [16–20].

The purpose of this review is to summarize our clinical experience and our results regarding the morbidity and mortality observed after Fontan surgery.

## Methods

304 patients who underwent Fontan surgery in our pediatric cardiovascular surgery unit of Florence Nightingale Hospital between 1995 and 2022 were included in the study. The patients were evaluated in pediatric cardiology and Fontan operation was decided by the surgical council. All of the patients were evaluated in our pediatric cardiology and pediatric cardiovascular surgery council in our hospital. The information of the patients was scanned retrospectively from the records in the hospital database.

Of the 304 patients included in our study, 26 patients underwent classical Fontan or lateral tunnel surgery (LT), and 278 patients underwent extracardiac Fontan(ECC) surgery. Of 304 cases, 218 had single ventricular pathology, 86 had complex cardiac pathologies with biventricular morphology but not amenable to biventricular repair.

Choussat and Fontan proposed 10 criteria for the success of Fontan surgery and for reducing the risk. Choussat and Fontan criteria [21]: 1. The operation should be performed between the ages of 3–15, 2. The heart rhythm is sinus rhythm, 3. Systemic venous return is normal, 4. Right atrial volume is normal, 5. Mean pulmonary artery pressure is ≤ 15 mmHg, 6. Pulmonary arterial resistance < 4 U/m^2^, 7. Pulmonary artery diameter / Aorta diameter > 0.75, 8. Left ventricular ejection fraction ≥ 60%, 9. Mitral valve intact, 10. Problems related to previous pulmonary artery surgeries absence.

Our patients were evaluated by the pediatric cardiology and cardiovascular surgery council, and surgery was decided for the patients who met the criteria. Fontan surgery was performed with the decision of the pediatric cardiology council (26 months, 29 months and 31 months) in 3 patients who had the above parameters but were younger than 3 years old.

Currently, the most acceptable methods of Fontan connection are extracardiac conduit and lateral tunnel total cavopulmonary connection. In the lateral tunnel operation, the superior vena cava is connected directly to the pulmonary artery and the inferior vena cava is connected to the pulmonary artery through a channel (lateral tunnel tunnel). The lateral tunnel technique creates a perfect flow towards the pulmonary artery, preserves low pressure conditions in the coronary atrium and involves a low risk of injury to the atrioventricular node. The intra-extracardiac conduit Fontan modifications were performed with cardiopulmonary bypass (CPB), aortic cross-clamping, and cardioplegic arrest by using an oblique low right atriotomy to anastomose a beveled expanded polytetrafluoroethylene (ePTFE) conduit to the internal opening of the inferior vena cava (IVC) to the right atrium.

EC Fontan modifications were performed on the CPB in a standard manner with end-to-end anastomosis of an ePTFE channel to the split caudal end of the IVC. Number 20 and 22 Gore-tex (W. L. Gore & Associates, Flagstaff, AZ) ring-supported PTFE condiuits was used in all patients.

Fenestration was performed in only 6 patients, other patients did not require fenestration. A fenestration was created using a 5 mm aortic staple along the left lateral aspect of the canal immediately cranial to the IVC-conduit anastomosis, followed by anastomosis around the fenestration of the cardiac end of the split IVC as before. VEDP > 2 mm Hg, MPAP > 15 mm Hg, PVR > 2 Wood U/m^2^, moderate or more atrioventricular valve insufficiency, and moderate or more systemic ventricular dysfunction were detected in 6 patients who received Fenestrated Fontan. Prolonged pleural effusion was defined as the need for a chest tube for > 14 days [22].

Protein C, protein S, antithrombin III and factor VIII levels are evaluated in the follow-up of the risk of thromboembolism.

## Fındıngs

304 patients who had Fontan surgery between 1995 and 2022 were included in the study. The age range of our patients was 2–17 years (mean 5.3 years, standart deviation 3,42). 171 of the patients were male and 133 were female.

Classic Fontan or lateral tunnel surgery was performed on 26 patients between 1995 and 1998. Extracardiac Fontan surgery was performed with 20 or 22 Goretex in 278 cases between 1999 and 2022. The mean age of the patients at Fontan surgery was 5.3, and ranged from 2 to 17 years. The surgical procedures performed before Fontan surgery and the demographic information of the patients are shown in Table 1.

**Table 1:** Information of patients who have had Fontan surgery (n = 304)

Age (years)	5.3 ± 3.43 (2–17)
Male (n,%)	171 (56%)
Female (n,%)	133 (44%)
Dominant ventricular morphology (%)	Right ventricle (129, 42%)
	Left ventricle (172, 57%)
	Undetermined ventricle (3, 1%)
Primer Fontan (n,%)	11 (4%)
Fontan after BT shunt (n,%)	14 (5%)
Fontan after Glenn (n,%)	190 (62%)

218 of 304 cases were patients with single ventricular pathology. 86 cases were patients with two ventricular morphologies but complex cardiac pathology. Of the patients with complex cardiac pathology, 45 had corrected-TGA-VSD-PS, 12 had criss cross heart, 11 had DORV, 6 had CAVC-TOF, 3 had D-TGA-VSD-PS, 2 had IVS-pulmonary atresia, and the last 2 patient groups had hypoplastic tricuspid valves. The distribution of these pathologies is shown in Table 2.

**Table 2:** Pathologies of patients with biventricular morphology (n = 86)

	Dextrocardi situs ınversus	Dextrocardi situs solitus	Dextrocardi coronary anomalies	Levocardi situs solitus	Right atrial Isomerism	Left atrial ısomerism	Remote VSD	Coroner sinusoids	AV valve-straddling overriding pathologies
Corrected TGA-VSD-PS (n = 45)	11	7	–	24	2	1	–	–	–
Criss-cross-heart-VSD-PS (n = 12)	5	2	–	2	2	1	–	–	4
DORV (n = 11)	2	1		3	5	–	3	–	6
CAVC-TOF (n = 6)	1	1	–	–	3	1	–	–	2
D-TGA-VSD-PS (n = 3)	–	–	–	3	–	–	–	–	–
TOF-Hypoplastic tricuspid valve (n = 2)	–	–	–	2	–	–	–	–	–
IVS-Pulmoner atresie (n = 2)	–	–	–	2	–	–	–	–	–

The age range of these 86 cases was between 4 and 17 (mean 5.1 ± 1.5), 48 of them were boys and 38 of them were girls. The hospital stay ranged from 7 to 46 days (mean 13 days). The procedures applied to 86 patients with biventricular morphology before Fontan surgery are shown in Table 3.

**Table 3:** Procedures applied to patients with biventricular morphology before Fontan surgery and their information (n = 86)

Age at definitive procedure (years)	5.1 ± 1.5 (4–17)
Weight at definitive procedure (kg)	8.0 ± 2.2
Dominant ventricular morphology (n,%)	Left ventricle (48, 56%)
	Right ventricle (38, 44%)
Fontan after Glenn (n,%)	32 (37%)
Fontan after BT shunt (n,%)	2 (2%)
Fontan after BT shunt + Glenn (n,%)	52 (60.%)

In our follow-up, the mean follow-up period of our patients was 12 years (3 months–27 years).

Early and mid-term postoperative complications in our patients who underwent Fontan operation are shown in Table 4.

**Table 4:** Complications after Fontan surgery

	Classic Fontan (n = 26)	Extracardiac Fontan (n = 278)	*p* value
Mean hospital stay (days)	16	14	0.000
Median ICU stay (days)	3	2	0.255
Acut renal failure (n,%)	–	3 (1%)	0.596
Nodal rhythm (n,%)	1 (4%)	13 (5%)	0.847
Temporary neurological problem (n,%)	–	1 (1%)	0.760
Prolonged pleural drainage (n,%)	1 (4%)	21 (8%)	0.487
Hemorrhage revision (n,%)	–	4 (2%)	0.540
Protein-losing enteropathy (n,%)	–	2 (1%)	0.666
Mortality (n,%)	2 (8%)	2 (1%)	0.003

Postoperative morbidity and mortality of patients who underwent classical Fontan and extracardiac Fontan surgery were compared. Patients undergoing extracardiac Fontan operation mean hospital stay was 14.5 days and 16 days in patients treated with classical Fontan. There was a statistically significant difference between the two groups (*p* < 0.000).

Comparing the mortality rates between the 2 groups. Mortality was found to be low in Extracardiac Fontans (*p* < 0.004). When the two groups were compared in terms of length of stay in intensive care unit (ICU), acute renal failure, nodal rhythm, temporary neurologic problem, prolonged pleural drainage, hemorrhagiac revision, protein loosing enteropathy, there was no statistically significant difference (*p* > 0.05). The median pleural effusion time in patients who received extracardiac Fontan at our center was 15 (IQR 10.0–20.7) days.

Two patients (%2.3) who developed protein-losing enteropathy and associated hypoalbuminemia were controlled with medical treatment and physical exercises. Thrombo-embolism and graft thrombosis did not development 4 of our female patients got married and had children.

Fontan operation was performed on 86 patients with complex pathology unsuitable for biventricular repair. No hospital mortality was observed in this group. Two of our patients with single ventricular morphology died. The first patient to die had hemiparesis due to preexisting SVA. The patient, who could not be mobilized after surgery, died at the 3rd postoperative month due to sepsis developing after low cardiac output. The second patient died due to sudden cardiac arrest after postoperative pericardial tamponade treatment.

## Dıscussıon

The Fontan procedure was developed in humans in the early 1970s for situations where there are no two separate ventricles to pump blood in parallel to the pulmonary and systemic circulation [23]. However, it is currently used in functional single ventricular pathologies where biventricular repair is not. The progression of the procedures applied to patients with single ventricular morphology has been divided into 4 generations. The atriopulmonary Fontan Kreutzer procedure was used in the first generation, lateral tunnel FP in the second generation, extracardiac canal FP in the third generation, and fenestrated intra/extracardiac canal in the fourth generation.

Of the patients in our study, 218 had single ventricular pathology, 86 patients had functional single ventricular pathology despite having biventricular anatomy. Extra cardiac conduit FP in 276 patients, lateral tunnel FP was performed in 26 patients, and the intra/extracardiac conduit with fenestration was performed in 6 patients.

In addition to single ventricular condition, the biventricular approach is avoided in some cardiac malformations. Patients may have inadequate ventricles (for example hypoplastic left ventricle in Shone's syndrome) or AV valves (e.g. pulmonary atresia/tricuspid stenosis with unimpaired ventricular septum), c-TGA, unbalanced AV canal defect, DORV with unspecified VSD or heterotaxy syndromes with complex ventricular relationships.

In the pathologies of patients with a functional single ventricle; Syndromes of c-TGA, criss-cross heart, D-TGA-VSD-PS, TOF-hypoplastic tricuspid valve, pulmonary atresia with intact ventricular septum, unstable AV channel, DORV with unspecified VSD, or heterotaxy with complex ventricular association have been reported. The presence of cardiac positions (situs inversus/solitus and heterotaxy syndromes) accompanying these pathologies are similar to those written in the literature [24]. Consistent with the literature, dextrocardi-situs inversus was seen in 19 cases, dextrocardi-situs solitus in 11 cases, right atrial isomerism in 12 cases, left atrial isomerism in 3 cases.

Developments began in the early 1980s to achieve a successful Fontan circulation. Conclusions for children with a functional single ventricle were significantly developed with the modified Fontan operation. Ventricular function and well-developed pulmonary vascular bed are important factors affecting the prognosis for the Fontan circulation to work successfully [25–27]. Additional surgical procedures were performed to prepare patients for a well-functioning Fontan circulation and to ensure its long-term success. Hopkins and colleagues proposed bilateral cavopulmonary anastomosis before Fontan was completed. Before Fontan surgery in our patient population; Glenn shunt was applied to 190 patients, BT shunt + Glenn shunt to 89 patients and BT shunt to 14 patients. Surgery was performed before Fontan surgery in 86 patients with single ventricular function in our study.Glenn shunt was applied to 32 patients, Glenn + BT shunt to 52 patients, and BT shunt to 2 patients. The effect of additional procedures performed before the completion of the Fontan circulation on the patient's prognosis is very important. Palliative surgical operations for the preparation of the Fontan circulation, they lead to preparation of the pulmonary vascular bed and preservation of ventricular function [28, 29].

During stage 2 palliation, systemic-pulmonary artery shunts placed in stage 1 palliation are closed. Because the diastolic circulation steals blood from the coronary circulation, early closure of the systemic pulmonary artery shunt is important as it may lead to a decrease in the capasity of the future single ventricle. Compared to bT shunt, the Glenn procedure provides a better performance for hemodynamics and is a more effectual method for pulmonary gas exchange [30, 31].

In our study, BT shunt was applied to 103 (33%) patients, Glenn shunt and BT shunt were applied to 89 patients as palliative surgery before Fontan operation. Thus contributing to the development of the pulmonary artery. However, it can also cause an increase in pulmonary pressure.

Stage 3 Fontan procedure gave better results in patients with previous bilateral cavopulmonary anastomosis. In addition to the stage 2 palliation, we performed pulmonary artery patch augmentation, AV valve repair, and atrial septectomy procedures. Systemic-pulmonary artery shunts placed between them during stage 1 palliation should be closed after stage 2 palliation.

The most important reason for early closure of systemic pulmonary artery shunt is that it may lead to a decrease in the performance of the future single ventricle as diastolic flow steals blood from the coronary circulation. If we compare the bidirectional Glenn procedure with a BT shunt, we see that it is a more effective method of pulmonary gas exchange, and in addition, it provides better hemodynamic performance in patients.

The NHLBI-funded Pediatric Heart Network has followed a cohort of Fontan patients for nearly 20 years. A recent study of this cohort (Fontan 3) found that ventricular morphology had no effect on survival of the 373 patients (mean age 21 years) included. Large cohort studies concluded that ventricular morphology was not among the risk factors for morbidity and mortality observed after the Fontan procedure [32–34].

Marathe et al. examined whether the Fontan procedure applied to patients with biventricular morphology was better than the Fontan procedure applied to patients with single ventricular morphology. The group studied 1377 patients in the Australia-New Zealand Fontan (ANZFR) registry and have been followed for an average of 11.5 years since Fontan. 79 of the patients had biventricular anatomy. Comparing patients with single ventricular anatomy and patients with biventricular anatomy when mortality and postoperative complications were compared, they found that there was no significant difference between the groups. [35].

Rossi et al. In their study of ventricular morphology in patients undergoing the Fontan procedure, they concluded that having an additional ventricle did not significantly differ in morbidity or mortality. This retrospective study reviewed all patients who underwent the Fontan operation over a remarkably long period of 40 years. In contrast to the ANZFR study, 22% of the 210 patients met the criteria of biventricular Fontan. There was also good distribution of dominant ventricle type with 78 patients having a dominant right ventricle, 115 patients having a dominant left ventricle and 17 patients with an undetermined ventricle. The authors found no difference in early complications after Fontan or early death for those with biventricular Fontan in comparison to single ventricle Fontan [36].

In our study, 86 (28.2%) of 304 patients met the criteria for biventricular Fontan. There were 218 (71.8%) patients with single ventricle Fontan structure, 124 patients with dominant left ventricle, 91 patients with dominant right ventricle, and 3 patients with undetermined ventricle. In our study, when patients with biventricular Fontan were compared with single ventricular Fontan, no significant difference was found in terms of early complications and mortality when evaluated with ventricular morphology. In our study, 2 (0.71%) of our 278 patients who extracardiac Fontan operation with an died, and these patients had a single ventricular morphology. Mortality in extracardiac Fontanes was found to be lower than in classical Fontanes.

As a result of improvements in Fontan surgical procedure and improvements in intensive care conditions, a decrease in mortality rates was observed.The postoperative 5, 10, and 15-year survival rates were 86%, 81%, and 73%, respectively [37].

Long-term increase in systemic venous pressure is one of the important criteria for prognosis and causes many complications. Early causes of morbidity associated with the Fontan operation include pleural and pericardial effusions, low cardiac output, sinus node injury, and pulmonary and systemic venous obstruction [38].

Chronic pulsatile flow deprivation is a pathology that causes a progressive increase in PVR, endothelial function and nitric oxide release in the pulsatile circulation, a decrease in vascular healing and consequently deterioration of lung growth, and is one of the major risks that can be seen in all Fontan patients. Factors associated with long-term morbidity after Fontan surgery: progressive dysfunction of the atrioventricular connection, right atrial distension, pulmonary vein bed occlusion, thromboembolic attacks, worsening of cyanosis due to: presence of surgically induced communication (fenestration) development of collateral arteriovenous circulation (systemic and pulmonary), decreased exercise tolerance, cognitive impairments, protein-losing enteropathy, progressive liver failure, plastic bronchitis [39, 40].

The long-term increase in the systemic venous pressure seems to play an important role and is reflected in many complications that may appear, such as distension of the right atrium and coronary sinus, hepatic dysfunction and increased splanchnic venous pressure [41, 42].

One of the postoperative complications is sinus node dysfunction and chronotropic insufficiency, which is common in the early and late periods after Fontan surgery and has been shown to damage exercise tolerance [43–46]. Recently, chronotropic insufficiency has been shown to decrease peak exercise capacity, increase mortality and the need for hospitalization and transplantation. Avoiding atrial incisions and suture lines is thought to reduce the rate of atrial arrhythmia that increases over time. In ECC Fontan, excluding the atrium during surgery theoretically protects the atrial wall from distention and the risk of subsequent arrhythmias [47].

Heterotaxy syndromes, relative anomalies of the atrioventricular valve, and the lateral tunnel technique are risk factors in themselves for the development of arrhythmia. Lateral tunnel technique leads to the development of arrhythmias due to suture lines placed in the atrium [48]. Bradyarrhythmias have also been observed in patients treated with ECC [49]. A normal circulating tachycardia can increase pulmonary blood flow by up to 35% without changing the diameter of the pulmonary vessel impedance, but this mechanism is absent in Fontan patients.

One of the complications after Fontan operation is prolonged pleural effusion [22]. It has been observed that patients with pleural effusion have a prolonged hospital stay and an increased risk of infection, requiring additional postoperative procedures [50]. Among the conditions that cause postoperative pleural effusion, there may be many factors affected by hormonal, inflammatory and hemodynamic changes. Gupta et al. investigated risk factors for persistent pleural effusion in 100 patients undergoing extracardiac Fontan surgery other than the risk factors just listed; Prolonged pleural effusion occurred in 37% of this group [22, 51]. Based on the definition of permanent pleural effusion lasting more than 14 days, Lo et al. showed that one-third of patients had persistent pleural effusion. Risk factors for prolonged pleural effusion include high pulmonary artery pressure and pulmonary atresia observed before Fontan operation. The increase in hydrostatic pressure in the Fontan circulation results from increased pulmonary vascular resistance, which is exacerbated by the absence of atrioventricular synchrony. It is predicted that prolonged exposure to high CVP may chronically affect the hydrostatic pressure, leading to a tendency to develop aortopulmonary collateral and venous collateral. Meyer et al. [52] reported that they applied fenestration to 90% of 160 Fontan patients, with a mean chest tube drainage time of 2 days and an average length of stay of 6 days. Fu et al. showed that absence of fenestration, low preoperative oxygen saturation, and postoperative infections are independent risk factors for long-term pleural drainage [53].

Extracardiac channels, acceptable aortic cross-clamping and cardiopulmonary bypass periods can be applied to prevent the development of pleural effusion. Use of inotropes and vasodilators when necessary for optimal intravascular volume with modified ultrafiltration and early postoperative extubation were recommended.In our study, prolonged pleural effusion was detected in 21 (7.6%) of 278 patients who underwent Fontan surgery with an extracardiac conduit.

Thromboembolic events after Fontan surgery significantly increase morbidity [54]. Normally, there was no reported difference for thrombotic events between LT and ECC [46, 55]. However, a recent report by Deshaies et al. [56] showed that in a highly variable time analysis of 522 patients across 12 institutions, ECC and LT had a similar arrhythmic risk and that ECC was associated with a lower incidence of thromboembolic events. The clinical incidences of the above-mentioned conditions vary through different studies from 3% up to 16% for venous thromboses and from 3% up to 19% for vascular strokes [57–59].

Most causes of early ECC deaths were attributed to thromboembolic complications, although there was no overall difference in thromboembolic events after Fontan operation between the groups. It is difficult to explain whether these complications are caused by the prosthetic material used in ECC or by the preoperative thrombosis tendency of these patients. These complications are frequently seen in the first 32 months after surgery, but attacks have also been reported in patients more than 16 years after surgery. Although patients use different doses of aspirin, warfarin and heparin in the literature, thromboembolic attacks still continue.Daily administration of aspirin (80 mg) is probably a convenient, safe and effective medical regimen [55].

Protein-losing enteropathy causes protein loss through the gastrointestinal tract and is associated with significant mortality rates, occurring weeks to years after Fontan surgery [60, 61]. Although the exact causes of its formation are unknown, increased venous pressure in the splanchnic region and postoperative low cardiac output have been considered as etiological factors.There are 3 main causes of protein-losing enteropathy. These are chronic venous congestion, impaired intestinal lymphatic drainage and intestinal inflammation. Clinical manifestations are related to the degree of hypoproteinemia, and in addition, ascites, peripheral edema, pleural effusion, immunodeficiency, and coagulation disorders may occur. The increase of α1-antitrypsin in feces confirms enteric protein loss [62, 63].

Fenestration, pacing, or even transplantation may be needed. Pharmaceutical treatment includes the administration of diuretics and dietary supplements. Corticosteroids and heparin have been empirically proved to reduce the protein loss in some patients [64, 65]. However, the prognosis of protein-losing enteropathy remains poor.

Kreutzer et al. reviewed the fifty-year results of the Fontan-Kreutzer procedure and grouped the factors that compromised late results into three categories. He noted that the suboptimal surgical approach, ventricular dysfunction and increases in PVR will help identify high-risk candidates for the Fontan operation, with Coussat's "ten commandments" or the Birmingham-England group's "two orders" in the modern age of congenital heart surgery. Along with ventricular function, MPAP, transpulmonary gradient, and PVR are the most important parameters to address the long-term outcome of the Fontan circulation [66, 67].

## Conclusion

The results of this study showed that during 12 years of follow-up, the overall survival and the functional status of the survivors after an extracardiac Fontan procedure were satisfactory. The incidence of late death, reoperation, obstruction of the cavopulmonary pathway, arrhythmias, and thromboembolism was low. There are unresolved questions regarding the choice of Fontan procedures and their impact on complication development. It is important to combine the experiences of large series in finding the answers to these questions. In our study, we wanted to contribute to the literature by presenting our 27 years of experience in Fontan surgery.

## Data Availability

The datasets used and analyzed during the current study are available from the corresponding author on reasonable request.

## Abbreviations

TGA: Transposition of the great arteries
VSD: Ventricular septal defect
PS: Pulmonary stenosis
IVS: Intact ventricular septum
IVC: Inferior vena cava
CAVC: Complete AV canal connection
D-TGA: Dextro transposition of great arteries
DORV: Double outlet right ventricle
FP: Fontan procedure
ECC: Extracardiac conduit
LT: Lateral tunnel
TCPC: Total cavopulmonary connection
CPB: Cardiopulmonary bypass
ePTFE: Expanded polytetrafluoroethylene
CVP: Central venous pressure
MPAP: Mean pulmonary artery pressure
PVR: Pulmonary vascular resistance
SVA: Cerebrovascular disease
ICU: Intensive care unit
